# Virological Response and Muscular Adverse Events during Long-Term Clevudine Therapy in Chronic Hepatitis B Patients

**DOI:** 10.5812/hepatmon.6056

**Published:** 2013-04-01

**Authors:** Byung Kook Kim, Soon Young Ko, So Young Kwon, Eugene Park, Jeong Han Kim, Won Hyeok Choe, Chang Hong Lee

**Affiliations:** 1 Department of Internal Medicine, Konkuk University School of Medicine, Seoul, 143-729, Korea

**Keywords:** Hepatitis B, Chronic, 2'-fluoro-5-methylarabinosyluracil, Response Elements, Adverse Effect, Hepatitis B, Chronic, 2'-fluoro-5-methylarabinosyluracil, Response Elements, Adverse Effect

## Abstract

**Background:**

Recently, several reports issued clevudine induced myopathy in the long term use.

**Objectives:**

The aim of this study was to investigate antiviral effects and adverse events of clevudine monotherapy in patients with chronic hepatitis B (CHB).

**Patients and Methods:**

The subjects were 110 treatment-naïve CHB patients. They were treated with 30 mg clevudine/day for more than six months. Virological and biochemical tests, including that for serum creatine kinase (CK), were monitored at baseline and at 3-month intervals during treatment period.

**Results:**

In HBeAg-positive patients, the cumulative rates of virological response were 74.0 %, 68.5 %, and 67.3 % after one, two, and three years of clevudine treatment, respectively. Cumulative rates of HBeAg loss or seroconversion were 17.8 %, 30 %, and 31.5 % after one, two and, three years of clevudine treatment, respectively. In HBeAg-negative patients, the cumulative rates of virological response were 97.3 %, 100 %, and 94.6 %, respectively. Virological breakthrough occurred in 27 patients. The rtM204I mutation in HBV polymerase was predominantly detected. Muscular adverse events were observed in 15 patients. All patients with myopathy recovered after the cessation of clevudine monotherapy. Fluctuations in CK level during the clevudine treatment period were frequently observed irrespective of development of myopathy. Multiple episodes of CK elevation were significantly related to the development of myopathy.

**Conclusions:**

Long-term clevudine monotherapy is effective for suppression of serum HBV DNA level and normalization of serum alanine amino transaminase levels, but associated with occurrence of rtM204I mutation. Clevudine-induced muscular adverse events are not uncommon, although they are totally reversible after cessation of the treatment. Muscular adverse events and serum CK level should be carefully monitored during long-term treatment with clevudine.

## 1. Background

Treatment of chronic hepatitis B virus (HBV) infection with nucleoside/nucleotide antiviral agents aims for the prolonged suppression of viral load and prevention of progress to decompensated liver cirrhosis and hepatocellular carcinoma. To achieve this goal, the antiviral agent should be a potent suppressor with sustained effects, and should be safe for long-term use (1). Clevudine [L-FMAU, (1-(2-fluoro-5-methyl-β, L-arabinofuranosyl) uracil)] is a pyrimidine L-nucleoside analog with potent and sustained antiviral activity against HBV (2-4). It is an enantiomer of D-FMAU and is structurally related to fialuridine, lamivudine, and telbivudine (2). Phase II studies on clevudine therapy found that the median reduction in serum HBV DNA level from baseline to week 12 of treatment was 4.5 log10 copies/ml and post-treatment antiviral activity was sustained for 12 weeks after therapy without any significant complication (3, 4). In phase III clinical trials on clevudine therapy, the median serum HBV DNA reductions were 5.1 log10 copies/ml in hepatitis B e antigen (HBeAg)-positive patients and 4.2 log10 copies/ml in HBeAg-negative patients after 24 weeks of treatment (5, 6). Clinical trial of one-year clevudine treatment demonstrated that clevudine has a potent antiviral efficacy and low incidence of resistance (7-9). Viral breakthrough during clevudine therapy occurs less frequently than with lamivudine; the incidence of viral breakthrough at 48 weeks of clevudine treatment was 9.4 % (8). However, data on the efficacy and safety of this antiviral agent during its long-term use are limited. Recent clinical trials of long-term therapy with clevudine in the USA were terminated because of safety issues after several studies found that it induced myopathy (10-12). The discontinuation of clevudine therapy appears to be sufficient to manage this myopathy and enable recovery from it. Lack of clinical experience with this drug-related toxicity makes it difficult to detect and identify similar potential adverse events in future drug development. Muscle enzymes such as creatine kinase (CK), lactate dehydrogenase, and lactate could be considered as screening tools to diagnose myopathy. However, the muscular adverse events during long-term clevudine treatment have not yet been clearly identified.

## 2. Objectives

The present study was thus conducted to assess virological response and muscle-related adverse events associated with long-term monotherapy with clevudine.

## 3. Patients and Methods

### 3.1. Patients

The study subjects were 110 chronic hepatitis B (CHB) patients who were enrolled consecutively between January 2007 and June 2009 at Konkuk University Medical Center. Hepatitis B surface antigen (HBsAg) had been detectable in the serum of all enrolled patients for more than six months, their serum alanine amino transaminase (ALT) level was greater than twice the upper limit of normal (ULN) for chronic liver disease, and they had not been treated previously with anti-HBV nucleosides or nucleotides. Those with HBeAg-positive CHB exhibited a serum HBV DNA level greater than 5 log10 copies/ml, while in those with HBeAg-negative CHB and/or with compensated cirrhosis, it was greater than 4 log10 copies/ml. Exclusion criteria included patients with chronic renal failure, decompensated liver disease, and co-infection with hepatitis C, hepatitis D, and HIV. Pregnant or breastfeeding women and women of child-bearing age who were unwilling to use barrier contraceptive devices were also excluded. Cirrhosis was diagnosed clinically based on imaging findings (abdominal computed tomography or magnetic resonance imaging) and compatible clinical features (esophageal varices or thrombocytopenia). The study protocol was approved by the Institutional Review Board of Konkuk University Medical Center (KUH 1010277). All of enrolled patients gave their written, informed consent for storing their sera and for the biochemical tests and HBV genetic studies.

### 3.2. Study Design

All 110 treatment-naïve patients received 30 mg of clevudine once daily for more than six months from January 2007 onwards. They were monitored at baseline and every three months during clevudine therapy. They were assessed clinically by obtaining their medical history, and subjecting them to a physical examination, electrocardiography, and testing of blood HBeAg status (Cobas e immunoassay, Roche, Switzerland) and serum HBV DNA levels. At the three-monthly follow-ups during the treatment period, the patients were assessed clinically to determine their tolerability, and subjected to a physical examination, blood chemistry, and HBV status testing. Serum HBsAg levels were tested after 12 months of therapy. Serial blood samples were collected and stored frozen at -80℃ from each patient at the time of initiation of each antiviral agent, every three months during treatment, and at the time of viral breakthrough. Serum HBV DNA levels were assessed using the COBAS Amplicor polymerase chain reaction (PCR) assay, which has a lower limit of detection of 300 copies/ml (Roche Molecular Systems, Branchburg, NJ, USA).

### 3.3. Virological Response

Primary non-response was defined as a decrease in serum HBV DNA to less than 2 log10 copies/ml at six months therapy (13). Primary treatment failure was considered to have occurred when serum HBV DNA levels remained above 5 log10 copies/ml through month 12. Virological response was defined as a decrease in the serum level of HBV DNA to a level undetectable by PCR assay, or the loss or seroconversion of HBeAg in patients who were initially HBeAg positive. Biochemical response was defined as a decrease in serum ALT level to within the normal range. Complete response was defined as fulfilling the criteria of biochemical and virological response and loss of HBsAg. Virological breakthrough was defined as an increase in serum HBV DNA to more than 1 log10 copies/ml above the nadir after achieving a virological response during treatment. When viral breakthrough developed during the treatment period, we tested a restriction fragment mass polymorphism (RFMP; Genematrix, Youngin, Korea) at rt180 and rt 204 in HBV polymerase gene (14). Adverse events and laboratory abnormalities related to myopathy were monitored throughout the follow-up period and graded for severity according to criteria adapted from the Division of Acquired Immunodeficiency Syndrome, National Institute of Allergy and Infectious Diseases (15). Clevudine-induced myopathy was diagnosed according to clinical symptoms of proximal muscle weakness causing inability to perform usual social and functional activities and corresponding neurological and physical examination findings by an expert neurologist. Laboratory findings related to muscle damage and electromyography (EMG) were used as ancillary data. Types of myopathy were classified pathologically by muscle biopsy (16). Grade 3 aminotransferase elevations were defined as 5.1–10.0 times the baseline; grade 4 elevations were defined as ≥ 10 times the baseline and/or evidence of hepatic failure. We measured levels of serum CK every three months during the clevudine therapy period using by stored frozen sera at -80℃. Grades 1-2 CK elevations were defined as levels 3.0-9.9 times the ULN; grade 3-4 elevations were defined as ≥ 10 times the baseline.

### 3.4. Statistical Analysis

All data were entered into a database and analyzed using SPSS 13.0 for Windows software (SPSS, Chicago, IL, USA). Continuous variables are presented as the mean ± SD values, and categorical variables as absolute and relative frequencies. Serum HBV DNA level and virological breakthrough were analyzed by t-test. The incidence of undetectable levels of serum HBV DNA levels and virological breakthrough were analyzed using Pearson’s 2 tests. P values less than 0.05 were considered statistically significant.

## 4. Results

### 4.1. Baseline Characteristics of the Patients

A total of 110 patients were studied. The baseline characteristics of patients are summarized in Table 1. The mean age of the enrolled patients was 45.1 years, 74 patients (67.3 %) were HBeAg positive, and 36 had cirrhosis. The mean follow-up duration was 17.3 months. The median serum HBV DNA level at baseline was 7.84 log10 copies/ml in the HBeAg-positive patients and 6.43 log10 copies/ml in the HBeAg-negative patients. Nine patients (8.2 %) had been taking an oral statin, a well-known cause of drug-induced myopathy. There was no demonstrable concurrent medication such as corticosteroids, chloroquine, hydroxychloroquine, certain 3-hydroxy-3-methylglutaryl-coenzyme reductase inhibitors, fibric acid derivatives, penicillamine, zidovudine, cyclosporine, erythromycin, niacin, and/or azole antifungals (Table 1).

**Table 1:** Baseline Characteristics of the Patients

	HBeAg-positive patients (n = 74)	HBeAg-negative patients (n = 36)	Total (n = 110)
**Years, y, Mean (Range)**	41.7 (24-74)	52.1 (29-73)	45.1(24-74)
**Male, No. (%)**	50 (67.6)	19 (52.8)	69 (62.7)
**Liver cirrhosis, No. (%)**	18 (24.3)	18 (50)	36(32.7)
**Hepatocellular carcinoma, No. (%)**	5 (6.8)	4 (11.1)	9(8.2)
**Fatty liver, No. (%)**	18(24.3)	7(19.4)	25(22.7)
**Co-morbidity**			
Alcohol intake > 20g/day, No. (%)	26 (35.1)	10 (27.8)	36 (32.7)
Hypertension, No. (%)	8 (10.8)	9 (25.0)	17 (15.5 )
Diabetes mellitus, No. (%)	3 (4.1)	6 (16.7)	9 (8.2)
Thyroid or rheumatic disease, No. (%)	2 (2.7)	1 (2.8)	3 (2.7)
**Concurrent medications, No. (%)**	23 (31.1)	21 (58.3)	44 (40)
Drug-related myopathy a, No. (%)	5 (6.8)	4 (11.1)	9 (8.2) b
**Serum ALT, IU/l**			
Mean ± SD	235.3 ± 199.5	197.2 ± 150.8	222.8 ± 185.2
Median (Range)	153.3 (28-871)	153.0 (25-618)	153.2 (25-871)
**Serum HBV DNA (log10 copies/ml)**			
Mean ± SD	7.73 ± 1.27	6.29 ± 1.17	7.25 ± 1.41
Median (Range)	7.84 (5.03-9.96)	6.43 (3.60-8.12)	7.34 (3.60-9.96)
**Follow period, ** **mo**			
Mean ± SD (Range)	21.4 ± 9.5 (6-48)	26.1 ± 10.0 (6-48)	22.9 ± 9.9 (6-48)

Abbreviations: ALT, alanine aminotransferase; HBV, hepatitis B virus; SD, standard deviation

^a^Corticosteroids, chloroquine, hydroxychloroquine, certain 3-hydroxy-3-methylglutaryl-coenzyme (HMGCoA) reductase inhibitors, fibric acid derivatives, penicillamine, zidovudine, cyclosporine, erythromycin, niacin, and/or azole antifungals

^b^8.2 % of patients were taking a statin orally

### 4.2. Virological and Biochemical Response

**Figure 1:**
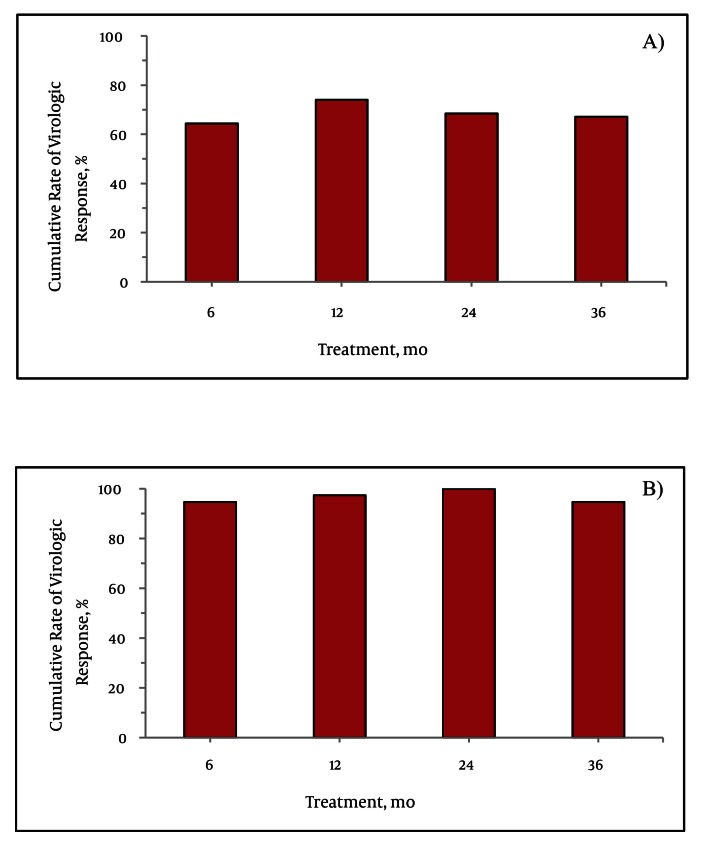
Cumulative Rates of Virological Response in: A) HBeAg-Positive and B) HBeAg-Negative Patients

**Figure 2:**
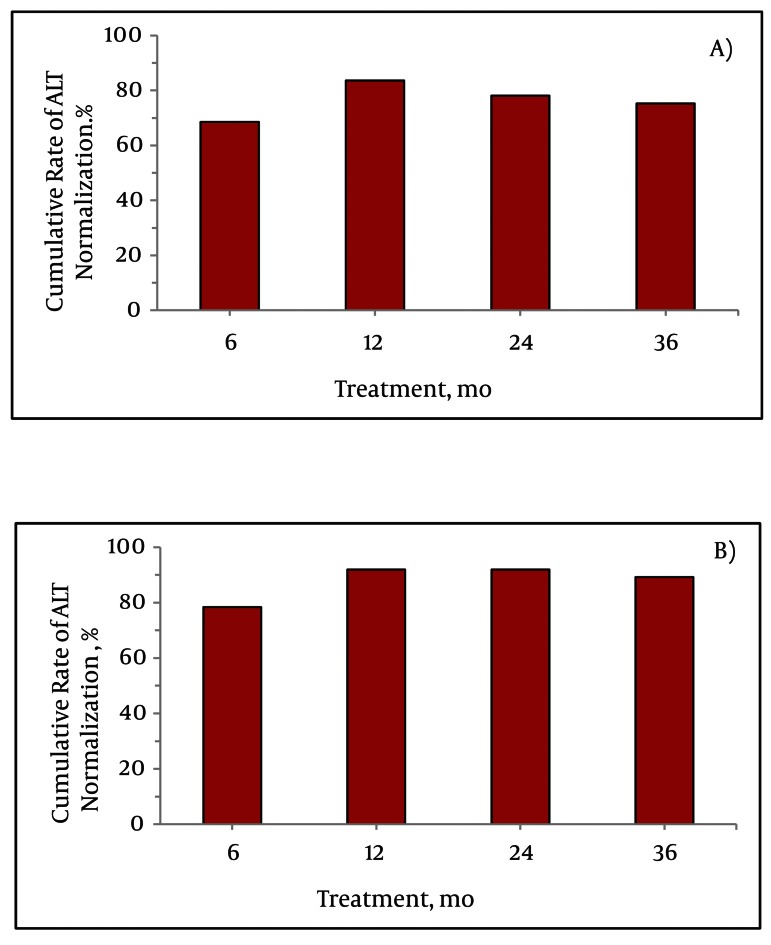
Cumulative Rates of Biochemical Response in: A) HBeAg-Positive and B) HBeAg-Negative Patients

Of HBeAg-positive patients, serum HBV DNA was undetectable in 64.4 %, 74.0 %, 68.5 %, and 67.1 % at six, 12, 24, and 36 months of clevudine treatment, respectively; the corresponding values for HBeAg-negative patients were 94.6 %, 97.3 %, 100 %, and 94.6 % (Figure 1). There were no primary non-responders or primary treatment failures in either of these two groups. HBeAg loss or sero conversion was observed in 17.8 %, 30.1 %, and 31.5 % at 12, 24, and 36 months of treatment, respectively. None of the patients experienced HBsAg loss or sero conversion during the treatment period. Among the HBeAg-positive patients, ALT level became normalized in 83.6 %, 78.1 %, and 75.3 % at 12, 24 and 36 months, respectively; the corresponding values for the HBeAg-negative patients were 91.9 %, 91.9 % and 89.2 % (Figure 2).

### 4.3. Virological Breakthrough and Genotypic Mutations

Viral breakthrough occurred in 27 patients during the follow up period. The cumulative incidence of viral breakthrough in HBeAg-positive patients was 11.8 % and 22.7 % at 12 and 24 months of therapy, while that in HBeAg-negative patients was 9 %, 23 %, and 24.5 % at 12, 24, 36 months, respectively. Baseline serum HBV DNA levels were 7.1 ± 1.4 log10 copies/ml and 6.4 ± 1.2 log10 copies/ml in the groups that did not and did develop viral breakthrough, respectively; the difference was not statistically significant (*P* = 0.345). Thus, initial serum HBV DNA level was not a risk factor for developing viral breakthrough. The rate of undetectable levels of HBV DNA at six months of treatment is significantly lower in the group that did not develop viral breakthrough (*P* = 0.001). Other factors, such as age, sex, HBeAg status, cirrhosis, underlying disease, fatty liver, and follow-up duration, were not correlated with developing viral breakthrough. Virological resistance was examined by RFMP for rt204 and rt180 at the time of virological breakthrough in 25 patients. The rtM204I mutation in the HBV polymerase gene was detected in 24 patients; the remaining patients had wild-type rt204 and rt180. Of 24 cases with the rtM204I mutation, three had co-dominant rtM204I and rtL180M mutations. One patient had a mixed pattern of rtM204I/V and rtL180M mutations.

### 4.4. Safety and Muscular Adverse Events

Patients underwent routine check-ups every three months during clevudine therapy to identify any adverse events and to evaluate therapeutic responses. The only clinically significant adverse event that led to treatment interruption or discontinuation during the treatment period was myopathy. None of the patients has experienced toxic-grade serum creatinine [≥ 1.1 times the ULN], rhabdomyolysis, or hepatic failure during treatment. Myopathy was diagnosed in 15 patients during the treatment period; the clinical and laboratory data of the patients during the evaluation of myopathy are summarized in Table 2. One patient had been taking 10 mg of atorvastatin once daily, a well-known cause of drug-induced myopathy. Of 15 patients with myopathy, eight were female, and six had cirrhosis. The mean duration of clevudine therapy before symptom onset was 14.0 months (n = 15; range, 9.3-23.5 months). Most of these patients complained about general weakness and difficulty in climbing the stairs. Weight loss was also an important finding of myopathy. EMG findings were variable. Only four of these patients also exhibited both grade 1 and 2 CK elevations. In one patient, examination of a biopsy specimen taken from the left vastus lateralis muscle revealed pathologic features compatible with myopathy. All patients with myopathy fully recovered from the condition after stopping clevudine or switching to another antiviral agent (0.5 mg/day entecavir). The mean recovery time after cessation of clevudine was 4.2 months (n = 14; range, 1.6-8.7). Serum CK levels were monitored in 99 patients. Grade 3/4 ALT or AST elevations during treatment were observed in only one case. CK elevation was frequently observed in the clevudine-treated patients. A total of 63 patients developed abnormal CK levels (i.e., more than the ULN) at least once during the follow-up period. Twenty four episodes of toxic-grade CK elevations (≥ 3.0 times the ULN) developed in 19 patients. Severe grades (grade 3/4, ≥ 10 times the ULN) of CK elevations developed in a patient at nine months of treatment, who had not developed myopathy. 100% in myopathy group and 57.1 % in non-myopathy group showed at least one episode of abnormal elevation of CK (*P* = 0.001). 93.3 % in myopathy group and 40.5 % in non-myopathy group showed at least two episodes of abnormal elevation of CK (*P* = 0.001). 40.0 % in myopathy group and 15.5 % in non-myopathy group showed at least one episode of toxic grade above 1 of CK (*P* = 0.026). No patient of both groups showed toxic grade above 3 of CK. There were frequent fluctuations in CK level during clevudine treatment in both groups. Hematologic and biochemical factors were not associated with developing myopathy during clevudine therapy (Table 3). Risk factors of developing myopathy were underlying thyroid or rheumatic diseases (*P* = 0.048) and abnormal episodes of CK elevation (*P* = 0.006) including toxic grade 1 or 2 CK elevations (*P* = 0.008) (Table 3).

**Table 2:** The Clinical Manifestations and Laboratory Data of Clevudine Induced Myopathy

	Patient 1 a	Patient 2	Patient 3	Patient 4	Patient 5	Patient 6	Patient 7	Patient 8	Patient 9	Patient 10	Patient 11	Patient 12	Patient 13	Patient 14	Patient 15
**Age, y/Sex**	46/F	53/F	36/F	40/M	37/M	46/M	27/F	67/F	45/F	45/M	74/F	40/M	31/M	61/M	51/F
**Diagnosis of liver disease**	LC/HCC	LC	CHB	LC	CHB	CHB	CHB	LC	LC	CHB	CHB	CHB	CHB	LC	CHB
**Initial HBeAg status**	positive	positive	Negative	positive	positive	positive	positive	negative	negative	positive	positive	positive	positive	negative	negative
**Initial HBV DNA, log10 copies/ml**	6.20x10^5^	2.65x10^5^	4.72x10^3^	2.04x10^8^	2.09x10^8^	3.72x10^8^	1.05x10^8^	2.64x10^6^	3.52x10^6^	1.89x10^5^	1.05x10^8^	1.50x10^9^	1.30x10^7^	6.00x10^6^	2.20x10^7^
AST/ALT, U/L (0-40)	59/39	35/30	96/298	32/46	96/231	57/92	118/196	100/67	120/152	83/116	89/98	223/411	69/97	78/146	265/404
**Daily CLV dose, mg**	30	30	30	30	30	30	30	30	30	30	30	30	30	30	30
**Treatment outcome**															
Virological response	Yes	Yes	Yes	Yes	Yes	No	Yes	Yes	Yes	Yes	Yes	Yes	Yes	Yes	Yes
**Duration of CLV therapy before symptom onset, mo**	10.4	10.4	12.2	9.3	11.9	14.4	16.3	17.5	10.2	13.1	17	23.5	23.7	19.1	11.2
**Interval between symptom onset and evaluation, mo**	2.4	2.2	9.3	3.2	2.9	6.2	0.9	2.9	4.5	1.9	2.0	0.6	1.0	1.0	0.5
**Muscular symptom**															
Weight loss	No	Yes	No	Yes	Yes	Yes	No	No	No	Yes	No	Yes	Yes	Yes	No
General weakness	Yes	Yes	Yes	Yes	Yes	Yes	Yes	Yes	Yes	No	Yes	No	Yes	No	Yes
Fatigue	No	Yes	No	No	Yes	Yes	No	No	No	Yes	No	Yes	No	No	Yes
Hard to climbing stairs	Yes	Yes	Yes	Yes	Yes	Yes	No	Yes	Yes	Yes	Yes	Yes	No	Yes	Yes
Myalgia	No	Yes	No	No	No	No	No	No	Yes	No	No	No	Yes	No	No
Cramp	No	No	No	No	No	No	No	Yes	No	No	No	No	No	No	No
**Distribution and grade of weakness**															
Bulbar	No	Yes	No	No	No	No	No	No	No	No	No	No	No	No	No
Neck	5	4	4+	5	4+	4+	5	4+	4+	5	4	4+	4+	4+	4+
Upper extremities	4+	4+	4+	5	4+	4+	5	4+	4+	5	5	5	5	5	5
Low extremities	4	4-	4	4+	4+	4	4+	4	4+	4+	4	4	4	4	4
**EMG findings**	Chronic generalized	-	Active generalized	-	Active generalized	-	-	Mild inactive	Minimal active	-	Inactive	Subclinical, inactive	Inactive	-	Mild generalized
**Laboratory findings at the time of myopathy evaluation**															
CK,U/l (normal range:30-145)	526	904	2549	706	1388	435	638	2178	298	687	433	2565	223	162	429
AST/ALT,U/l (normal range < 40)	119/59	113/59	99/25	79/37	62/47	62/47	48/25	52/26	42/27	87/75	41/32	123/65	32/23	65/92	54/37
LDH,U/l (normal range:218-450)	1403	1277	715	614	1048	687	533	910	539	-	1086	973	613	637	633
**Rescue therapy**	ETV	ETV	None	ETV	none	ETV	none	ETV	ETV	none	ETV	none	none	none	ETV
**Recovery time, mo**	14	8.7	6.4	5.9	2.8	5.2	1.9	6.0	3.0	Follow-up loss	1.6	3.3	4.7	3.0	2.1

Abbreviations: ALT, alanine aminotransferase; AST, aspartate aminotransferase; CLV, clevudine; CK, creatine kinase; EMG, electromyography; ETV, entecavir; HCC, hepatocellular carcinoma; LC, liver cirrhosis; LDH, lactate dehydrogenase; P, positive; Pt, patient; N, negative

^a^This case was reported in our previous publication (28)

**Table 3:** Factors Associated With Developing Myopathy During Clevudine Therapy (Univariate Analysis)

	Non-myopathy (n= 95)	Myopathy (n= 15)	Odds ratio (95 % CI)	P value
**Age, y, Mean ± SD**	44.9 ± 11.76	16.6 ± 12.98		0.610^b^
**Male, No. (%)**	62 (65.3)	33 (34.7)	2.147 (0.716-6.443)	0.166^c^
**HBeAg-Positive, No. (%)**	64 (67.4)	10 (66.7)	0.969 (0.305-3.078)	0.957^c^
**Cirrhosis, No. (%)**	35 (36.8)	3 (20.0)	0.429 (0.113-1.624)	0.253^d^
**Hepatocellular carcinoma, No. (%)**	8 (8.4)	1 (6.7)	0.777 (0.090-6.696)	1.000^d^
**Fatty liver, No. (%)**	20 (21.1)	5 (33.3)	1.875 (0.575-6.111)	0.292^c^
**Alcohol intake (> 20g/day), No. (%)**	34 (35.8)	2 (13.3)	0.276 (0.059-1.296)	0.137^d^
**Hypertension, No. (%)**	16 (16.8)	1 (6.7)	0.353 (0.043-2.876)	0.459^d^
**Diabetes,** **No.** ** (%)**	8 (8.4)	1 (6.7)	0.777 (0.090-6.696)	1.000^d^
**Thyroid or rheumatic disease,** **No.** ** (%)**	1 (1.1)	2 (13.3)	14.462 (1.224-170.890)	0.048^d^
**Concurrent medications, No. (%)**	55 (57.9)	11 (73.3)	0.500 (0.148-1.685)	0.396^d^
**Concurrent medication related to myopathy a, No. (%)**	8 (8.4)	1 (6.7)	0.777 (0.090-6.696)	1.000^d^
**Serum CK**				
> ULN, No. (%)	31 (32.6)	4 (26.7)	5.677 (1.673-19.271)	0.006^c^
More than toxic grade 1, No. (%)	12 (12.6)	6 (40.0)	4.611 (1.393-15.268)	0.008^c^
Toxic grade 3 and 4, No. (%)	1 (1.1)	0 (0.0)	0.989 (0.969-1.010)	1.000^d^
**Initial serum HBV DNA**				
(log10 copies/ml), Mean ± SD	7.30 ± 1.39	6.98 ± 1.51		0.405^b^
**Initial serum ALT**				
(IU/l), Mean ± SD	232.5 ± 191.8	161.5 ± 124.1		0.169^b^

Abbreviations: ALT, alanine aminotransferase; CK, creatine kinase; HBV, hepatitis B virus; SD, standard deviation; ULN, upper limit of normal

^a^Corticosteroids, chloroquine, hydroxychloroquine, certain HMGCoA reductase inhibitors, fibric acid derivatives, penicillamine, zidovudine, cyclosporine, erythromycin, niacin, and/or azole antifungals

^b^Student’s *t*-test

^c^Pearson’s x^2^ test

^d^Fisher’s exact test

## 5. Discussion

This was an observational study of the efficacy and adverse events during long-term monotherapy with clevudine in treatment-naïve CHB patients. Clevudine monotherapy showed a potent antiviral effect within 12 months of treatment in both HBeAg-positive and HBeAg-negative patients. Phase III clinical trials of clevudine therapy demonstrated that 59.0 % of HBeAg-positive patients and 92.1 % of HBeAg-negative patients had undetectable levels of HBV DNA after 24 weeks of treatment (5, 6). Similarly, in the present study, 64.4 % of HBeAg-positive patients and 94.6 % of HBeAg-negative patients had undetectable levels of HBV DNA by quantitative PCR assay after six months of clevudine therapy. This study revealed a potent antiviral response at 12 months of treatment (74.0 % in HBeAg-positive and 97.3 % in HBeAg-negative patients), which is comparable to the results obtained for entecavir or tenofovir therapy (17, 18). Interestingly, there were no primary non-responders or primary treatment failures. However, the virological responses were lower at 24 and 36 months than that at 12 months, particularly among HBeAg-positive patients. The decreasing tendency of cumulative virological response reflects the development of antiviral resistance during long-term use of clevudine. Virological breakthrough occurred in 11.8 % and 22.7 % of the patients at 12 and 24 months of clevudine treatment, respectively, similar to the findings with telbivudine therapy (19). However, clevudine was associated with a higher incidence of virological breakthrough compared to entecavir and tenofovir (20, 21). Clevudine resistance is associated with rtM204I mutation in the HBV polymerase gene (22). In our study, genotypic mutation was detected in 24 out of 25 cases for which a genotype analysis was performed. Resistant mutations were rtM204I and/or rtM204V and/or rtL180M; these are well-known antiviral resistant mutations in HBV DNA against lamivudine and telbivudine (23). The structural similarity of clevudine and other two nucleoside analogues could thus cause development of the same antiviral-resistant mutation in HBV DNA polymerase. Nucleoside/nucleotide analogue-induced myopathy occurs during long-term treatment for chronic viral infection such as CHB and HIV (24). The following mechanisms of drug-induced myotoxicity have been proposed: (a) direct effects on a muscle organelle, such as mitochondria, lysosomes, and myofibrillar proteins, (b) alteration of muscle antigens, thereby inducing an immunological or inflammatory reaction, and (c) induction of systemic effects, such as electrolyte disturbances, nutritional deprivation, or malabsorption, which secondarily affect muscle function (16). The biological mechanisms of clevudine-related myopathy remained unclear. Recent studies of clevudine myopathy suggest mitochondrial dysfunction as a possible mechanism (11, 12). Our experience of emergence of myopathy in clinical practice was in 15 out of 110 CHB patients (13.6 %) receiving clevudine therapy lasting more than 12 months. There was no remarkable lactic acidosis and no permanent and irreversible myopathy damage in either this study or any of previously published case reports (unlike filauridine disaster) (25). Clevudine is known to possess prolonged potent antiviral activity after cessation of medication (6, 26). Moreover, the characteristic pharmacodynamic clearance of clevudine, which does not follow first-order kinetics as a function of time, may increase intracellular L-FMAU triphophate (L-FMAUTP) in long-term clevudine therapy. Therefore, the accumulation of intracellular L-FMAUTP levels could help exert an HBV inhibitory effect, but also cause drug-related toxicities under long-term therapy. Weight loss and general weakness were frequent subjective symptoms of myopathy. Difficulty in climbing the stairs is also a pathognomic symptom. The most common sign in our study was decreased motor function in proximal muscles. Asking the patient to complete an easy stair climbing is the best way of discriminating between myopathy and general weakness. The EMG findings of the myopathy patients were highly variable: active, inactive, acute, and chronic at the time of diagnosis. Clinical symptoms of myopathy and muscular power recovered gradually after discontinuation of clevudine in 14 of the followed-up patients. In 8 of these 14 followed-up patients, we switched from clevudine to entecavir, after which they all recovered completely from the clinical myopathic symptoms without experiencing viral breakthrough. Therefore, switching to another antiviral agent appears to be the best option for patients with antiviral-induced myopathy. The phase III GLOBE study found three symptomatic myopathic cases out of 680 patients who received telbivudine (19). That study also showed an adverse event, a grade 3 or 4 elevation in serum CK level that occurred in 7.5 % of patients receiving telbivudine. In the recent clinical studies of clevudine treatment, incidence of developing myopathy was reportedly higher than that for telbivudine, including our experience (15 %) (10, 12). There was no report about elevations of CK with clevudine. It is not possible to compare the toxic-grade elevation of CK levels between the GLOBE study and ours; because different criteria for grading the toxicity of CK were used (the criterion of toxic grade 3 of CK was defined as ≥ 10 times the ULN in our study, but as ≥ 7 times the ULN in the GLOBE study). Grade 3 or 4 elevations in CK level were not observed in our study. However, 64 % (63/99) of our patients experienced an abnormal CK level (i.e., more than the ULN) at least once through the follow-up period. In total, 24 episodes of toxic grade CK elevations developed in 19 patients. Elevation of serum CK level is not regarded as a specific marker for myopathy because CK can not only be elevated by myotoxic muscle damage, but also by various muscle injuries including trauma and injection, as well as other factors. In the clinical trials for telbivudine, CK elevations were transient and were not reliable markers of muscular adverse events (19, 27). However, our results showed that at least one or two episodes of abnormal CK elevation and at least one episode of toxic-grade CK > 1 occurred more frequently in patients developing myopathy. Elevation of CK levels before a diagnosis of myopathy could be a reflection of a subclinical stage of myopathy. For early detection of subclinical myopathy without overt muscle weakness, a careful medical examination should be followed by tests for serum CK level. Together, the findings of this study show that long-term clevudine therapy has potent antiviral effects and results in biochemical improvements. However, viral breakthrough was observed after 12 months of treatment. Clevudine-induced muscular adverse events were not uncommon during long-term clevudine treatment, even though the condition was totally reversible after cessation of the treatment or switching to a different antiviral medication. Monitoring of CK levels may be helpful for early detection of clevudine-induced myopathy during long-term therapy. Therefore, HBV DNA and muscular symptoms should be monitored closely during the long term use of clevudine.
